# Influenza-associated Deaths in Tropical Singapore

**DOI:** 10.3201/eid1201.050826

**Published:** 2006-01

**Authors:** Angela Chow, Stefan Ma, Ai Ee Ling, Suok Kai Chew

**Affiliations:** * Ministry of Health, Singapore;; † Singapore General Hospital, Singapore

**Keywords:** influenza, influenza A virus, excess mortality, death, tropical climate, Singapore, research

## Abstract

We used a regression model to examine the impact of influenza on death rates in tropical Singapore for the period 1996–2003. Influenza A (H3N2) was the predominant circulating influenza virus subtype, with consistently significant and robust effect on mortality rates. Influenza was associated with an annual death rate from all causes, from underlying pneumonia and influenza, and from underlying circulatory and respiratory conditions of 14.8 (95% confidence interval 9.8–19.8), 2.9 (1.0–5.0), and 11.9 (8.3–15.7) per 100,000 person-years, respectively. These results are comparable with observations in the United States and subtropical Hong Kong. An estimated 6.5% of underlying pneumonia and influenza deaths were attributable to influenza. The proportion of influenza-associated deaths was 11.3 times higher in persons age >65 years than in the general population. Our findings support the need for influenza surveillance and annual influenza vaccination for at-risk populations in tropical countries.

## Abstract 2

Surveillance and annual vaccination are needed for at-risk populations in tropical countries.

Influenza virus infections cause excess illness and deaths in temperate countries. In the Northern and Southern Hemispheres, influenza epidemics occur nearly every winter, leading to an increase in hospitalizations and deaths. The World Health Organization (WHO) estimated that these annual epidemics result in 3 to 5 million cases of severe illness and 250,000–500,000 deaths each year around the world (*1*). In the United States, influenza is responsible for 50 million illnesses and up to 47,200 deaths annually (*2**–**4*).

However, little is known about the impact of influenza on death rates in tropical regions, where the effect of influenza is thought to be less (*5*). In subtropical Hong Kong, deaths from underlying pneumonia and influenza attributable to influenza were estimated to be 4.1/100,000 population per year (*6*), higher than the rate (3.1/100,000) reported in the United States (*7*).

In tropical Singapore, influenza viruses circulate year round, with a bimodal increase in influenza incidence observed in April–July and November–January (*8**–**13*). The peaks correspond approximately to increased influenza activities in temperate countries in the Southern and Northern Hemispheres, respectively (*14**,**15*). Singapore is geographically located in the tropics, lying just north of the equator at latitude 1.5°N and longitude 104°E. Its climate is characterized by uniform temperatures of minimum 23°C–26°C and maximum 31°C–34°C and a relative humidity of 84% with maximum rainfall occurring in April and December (*16*). These conditions are typical for most tropical countries.

Any assessment of the true impact of influenza in the tropics must account for the more diffused seasonal pattern of influenza in the tropics and the cocirculation of other respiratory viruses. Respiratory syncytial virus (RSV) is also associated with excess deaths (*17*). Thus, the effect of this virus would have to be adjusted for. In this study, we used a regression model to examine the impact of influenza, by virus type and subtype, on deaths in a tropical country, while adjusting for potential confounding effects by other cocirculating influenza virus subtypes and RSV.

## Methods

### National Influenza Viral Surveillance

Influenza virus surveillance is carried out throughout the year and has been instituted in Singapore since 1973. We obtained monthly data on influenza A and B viruses and RSV from the WHO-designated National Influenza Centre in Singapore from January 1996 to December 2003. Specimens tested for influenza and RSV were obtained from pediatric inpatients at KK Women's and Children's Hospital, patients from Singapore General Hospital and other public-sector hospitals, as well as from adult outpatients with influenzalike symptoms treated at sentinel primary health clinics. Specimens were tested either with informed consent from patients for diagnostic purposes or as part of epidemiologic surveillance provided for by the Infectious Diseases Act.

RSV was detected by immunofluorescence tests and virus isolation. Influenza viruses were identified by direct antigen detection with immunofluorescence techniques, serologic tests with complement fixation, and virus isolation. To isolate influenza viruses, respiratory specimens were added to primary cynomolgus monkey kidney tissue cultures, which were rolled at 33°C and observed daily for cytopathic effects. If no effect was observed, the HeLa tubes were passaged blind at weekly intervals, and monkey kidney tissue cultures were tested for hemadsorption with guinea pig erythrocytes. Specimens were discarded after 4 weeks if negative. Influenza virus isolates were subsequently confirmed by immunofluorescence and typed by hemagglutination-inhibition tests using strain-specific antisera provided by the WHO Collaborating Centre for Influenza at the Centers for Disease Control and Prevention, Atlanta, Georgia, USA.

The National Influenza Center provided aggregated data for this study, i.e., monthly numbers of total respiratory specimens tested for influenza virus, positive influenza test results, and influenza virus isolates by subtype, as well as monthly RSV data. As the study spanned 8 years, we anticipated that positive results could be affected by changes in the number of tests performed. Therefore, we opted to use the monthly proportion of positive test results for a specific virus (with the respective monthly number of specimens tested for the specific virus as the denominator) as our indicator variable for virus activity, instead of monthly positive counts.

### Mortality Data

National mortality data were obtained from the Registry of Births and Deaths. Under the Registration of Births and Deaths Act, all deaths occurring within Singapore and its territorial waters are required to be registered within 3 days of the occurrence. Each death was categorized according to the International Classification of Diseases, 9th Revision (ICD-9) codes. In this study, death records were aggregated according to month of death from January 1996 through December 2003. Three death outcomes were analyzed: underlying pneumonia and influenza (P&I) deaths (ICD-9: 480–487), underlying circulatory and respiratory (C&R) deaths (ICD-9: 390–519), and all-cause deaths (ICD-9: 000–999).

### Statistical Methods

We first applied 6 negative binomial regression models (*18*) to the monthly number of deaths and monthly proportions of positive influenza virus and RSV tests, to examine the relationships between mortality and the respiratory viruses (namely, models 1–6). Details of the models are shown in the Appendix. We then used the full model (model 6) to obtain the relative risks (RR) of death (and 95% confidence interval [CI]) from influenza A, A (H3N2), A (H1N1), and B viruses, as well as RSV, for each of the 3 mortality categories (i.e., all-cause, underlying P&I, and underlying C&R deaths). We also attempted to estimate the excess number of deaths from the viruses.

Apart from accounting for possible overdispersion of the data in the models, the models also adjusted for potential confounding factors, including the number of days in each month, linear and squared term of time trend, seasonality (3–4 pairs of sine and cosine terms, allowing for 3 to 4 cycles per year to capture the main seasonal variations per year), temperature, and relative humidity. Linear and squared terms of time trend were included to capture secular trends, including population growth, changes in completeness of ICD coding, and changes in diagnostic methods. For each model, residuals were examined for discernible patterns and autocorrelation by means of residual plots and partial autocorrelation function plots. Since the unit of analysis was the calendar month, the lag effects of influenza and other covariates were not necessarily taken into account.

We estimated the influenza-associated mortality fraction by dividing the number of excess deaths (the difference between observed and expected deaths) by the number of observed deaths, when the proportion of positive influenza results was set to 0 in model 6. The 95% CI for each estimated fraction was obtained by using the bootstrap resampling method with 1,000 bootstrap resamples (*19*). The number of excess deaths attributable to influenza was then derived by multiplying the total number of deaths in each mortality category by the respective influenza-associated mortality fraction (*6**,**20*). We also derived the excess mortality rate per 100,000 person-years by dividing the number of excess deaths during the study period by the sum of the annual midyear population for the entire 8-year period. All analyses were performed by using S-Plus 6.0 Professional Release 2 software (Insightful Corporation, Seattle, WA, USA).

## Results

From January 1996 to December 2003, 57,060 specimens were tested for influenza virus, and 51,370 were tested for RSV. The volume of tests performed was noticeably lower in the first 2 years and in the last year of the study (Table 1). There were 9,103 positive results for RSV and 3,829 positive results for influenza. The annual mean number of tests positive for influenza A was 5.8% (range 2.6%–9.5%) and for influenza B, 0.9% (range 0.4%–1.6%). Annually, influenza A (H3N2) was the predominant influenza virus subtype in circulation. During the study period, peaks in influenza A (H3N2) were observed from December 1998 to January 1999 (the predominant circulating strain was A/Sydney/5/97), December 2000 to January 2001 (A/Moscow/10/99), December 2002 to January 2003 (A/Moscow/10/99), and October–November 2003 (A/Fujian/411/2002). Smaller peaks were noted in April–July in 1996, 1997, 1998, and 2002, and from September to November in 1999 and 2003.

**Table 1:** Annual influenza virus and respiratory syncytial virus (RSV) surveillance data, Singapore, 1996–2003

Year	Influenza virus							
	Influenza type*			Influenza A subtype†			RSV	
	No. specimens tested	Influenza A-positive test results (%)	Influenza B-positive test results (%)	No. specimens tested	A (H1N1)-positive isolates (%)	A (H3N2)-positive isolates (%)	No. specimens tested	Total positive test results (%)
1996	5,140	132 (2.6)	47 (0.9)	924	1 (0.1)	15 (1.6)	4,249	868 (20.4)
1997	5,255	208 (4.0)	39 (0.7)	1,041	9 (0.9)	17 (1.6)	4,441	902 (20.3)
1998	8,934	817 (9.1)	120 (1.3)	941	3 (0.3)	40 (4.3)	7,573	1,683 (22.2)
1999	7,548	714 (9.5)	74 (1.0)	1,001	1 (0.1)	99 (9.9)	6,915	1,004 (14.5)
2000	7,716	397 (5.1)	122 (1.6)	974	34 (3.5)	61 (6.3)	7,094	1,425 (20.1)
2001	8,171	300 (3.7)	76 (0.9)	1,023	33 (3.2)	44 (4.3)	7,445	1,415 (19.0)
2002	8,317	274 (3.3)	34 (0.4)	897	3 (0.3)	58 (6.5)	7,840	1,128 (14.4)
2003	5,979	454 (7.9)	21 (0.4)	1,130	6 (0.5)	121 (10.7)	5,813	678 (11.7)
Mean	7,133	412 (5.8)	67 (0.9)	991	11 (1.1)	57 (5.7)	6,421	1,138 (17.8)

*Respiratory specimens were tested for influenza by virus isolation, direct antigen detection, and serologic tests. †Influenza A isolates obtained from virus isolation were subtyped by using strain-specific antisera from the Centers for Disease Control and Prevention, Atlanta, GA, USA.

During the 8-year period, an annual mean of 15,616 deaths (range 15,301–16,024) occurred in Singapore. An average of 1,798 (range 1,545–2,340) underlying P&I deaths and 8,237 (range 7,833–8,715) underlying C&R deaths occurred each year (Table 2).

**Table 2:** Annual deaths in Singapore, 1996–2003*

Year	No. underlying P&I deaths (ICD-9: 480–487)	No. underlying C&R deaths (ICD-9: 390–519)	All-cause deaths (ICD-9: 000–999)
1996	1,690	8,420	15,569
1997	1,551	8,065	15,301
1998	1,781	8,286	15,649
1999	1,640	8,169	15,513
2000	1,795	8,253	15,691
2001	1,545	7,833	15,368
2002	2,077	8,158	15,811
2003	2,340	8,715	16,024

*P&I, pneumonia and influenza; C&R, circulatory and respiratory; ICD-9, International Classification of Diseases, 9th Revision.

The Figure shows the temporal trends for death outcomes as well as influenza virus and RSV activities. Peaks in monthly influenza A viruses corresponded very well with peaks in monthly all-cause deaths, underlying P&I deaths, and underlying C&R deaths.

**Figure:**
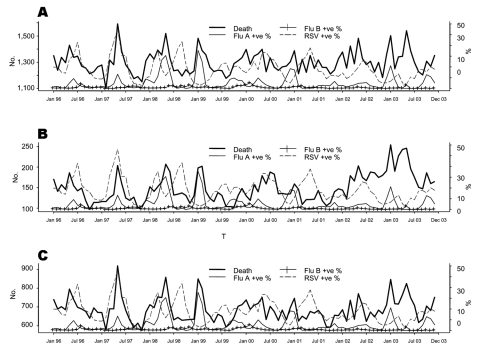
emporal trends in the positivity of specific respiratory viruses (influenza A, influenza B, and respiratory syncytial virus [RSV]) and the number of all-cause deaths (A), underlying pneumonia and influenza (P&I) deaths (B), and underlying circulatory and respiratory (C&R) deaths (C), January 1996–December 2003; +ve %, percent positive.

We tested the Spearman rank correlations between influenza and RSV, and meteorologic variables. Influenza A positivity (Spearman correlation [r] = 0.25) was weakly correlated with relative humidity. However, temperature (r = –0.71) was highly correlated with relative humidity. The influenza A (H3N2) subtype had a high correlation with influenza A (r = 0.75) (data not shown).

The relationship between deaths and each respiratory virus (influenza A, influenza B, and RSV) was examined by using a stepwise sequential approach (Table 3), i.e., first fitting each of the viruses into separate models (models 1–3), then adjusting for 1 of the other 2 viruses (models 4, 5), and finally, adjusting for all viruses in a single model (model 6). Furthermore, potential confounding factors were adjusted for and included in each model.

**Table 3:** Adjusted risk ratios* and p values for each 10% change in positive influenza A and RSV test results, and for each 1% change in positive influenza B† virus test results, 1996–2003‡

Mortality outcome/risk factor	Adjusted risk ratio (95% CI), p value					
	Model 1§	Model 2	Model 3	Model 4	Model 5	Model 6
All-cause deaths						
Influenza A	1.05 (1.04–1.06), 0.000	–	–	1.05 (1.04–1.06), 0.000	1.05 (1.04–1.06), 0.000	1.05 (1.04–1.06), 0.000
Influenza B	–	1.01 (1.00–1.02), 0.173	–	1.01 (1.01–1.02), 0.001	–	1.01 (1.01–1.02), 0.001
RSV	–	–	1.00 (0.99–1.00), 0.810	–	1.00 (1.00–1.01), 0.254	1.00 (1.00–1.01), 0.159
Underlying P&I deaths						
Influenza A	1.12 (1.08–1.16), 0.000	–	–	1.12 (1.08–1.16), 0.000	1.13 (1.09–1.17), 0.000	1.13 (1.09–1.17), 0.000
Influenza B	–	0.99 (0.96–1.02), 0.389	–	1.00 (0.94–1.03), 0.994	–	1.00 (0.98–1.03), 0.872
RSV	–	–	1.01 (0.99–1.02), 0.342	–	1.03 (1.00–1.02), 0.022	1.01 (1.00–1.02), 0.021
Underlying C&R deaths						
Influenza A	1.08 (1.06–1.10), 0.000	–	–	1.08 (1.07–1.10), 0.000	1.08 (1.06–1.11), 0.000	1.09 (1.07–1.11), 0.000
Influenza B	–	1.01 (0.99–1.02), 0.360	–	1.02 (1.01–1.03), 0.004	–	1.02 (1.01–1.03), 0.002
RSV	–	–	1.00 (0.99–1.01), 0.686	–	1.01 (1.00–1.01), 0.025	1.01 (1.00–1.01), 0.011

*Risk ratio estimates (95% confidence intervals) of each death category were adjusted for number of days in each month, linear and squared time trends, seasonal patterns, temperature and relative humidity; –, risk factor was not included in model. †Each 1% change was used for influenza B because of the small range of positive influenza B test results. ‡CI, confidence interval; RSV, respiratory syncytial virus; P&I, pneumonia and influenza; C&R, circulatory and respiratory. §Negative binomial regression models. Model 1, death outcome = influenza A + confounders; model 2, death outcome = influenza B (FluB) + confounders; model 3, death outcome = RSV + confounders; model 4, death outcome = model 1 + FluB; model 5, death outcome = model 1 +RSV; model 6, death outcome = model 4 + RSV.

Influenza A had significant and robust effects on monthly all-cause deaths (RR 1.05 for each 10% change in positive test results, without adjusting for influenza B virus, RSV, and other potential confounding factors; vs. RR 1.05, after adjusting for influenza B, RSV, and other confounding factors), underlying P&I (RR 1.12 vs. RR 1.13), and underlying C&R (1.08 vs. 1.09) deaths.

In Table 4, we used model 6 (as described in Table 3) to further explore the association between influenza A virus subtypes and the 3 death outcomes. We replaced influenza A variable with influenza A subtypes and adjusted for influenza B virus, RSV, and other confounding factors. Only influenza A (H3N2) had significant (all p values <0.001) effects on all-cause deaths (RR 1.04 for each 10% change in positive test results, 95% CI 1.02–1.05), underlying C&R deaths (1.05, 1.04–1.07), and underlying P&I deaths (1.08, 1.04–1.12).

**Table 4:** Association between influenza A virus subtypes and 3 death outcomes*

Model 6 mortality outcome	Adjusted risk ratio (95% CI), p value†			
	Influenza A (H1N1)	Influenza A (H3N2)	Influenza B	RSV
All-cause deaths	1.00 (0.96–1.04), 0.928	–	1.01 (1.00–1.02), 0.178	1.00 (0.97–1.00), 0.824
	–	1.04 (1.02–1.05), 0.000	1.01 (1.00–1.02), 0.008	1.00 (1.00–1.01), 0.484
Underlying P&I deaths	1.00 (0.88–1.13), 0.993	–	0.99 (0.96–1.02), 0.409	1.01 (0.99–1.02), 0.369
	–	1.08 (1.04–1.12), 0.000	1.00 (0.97–1.03), 0.878	1.01 (1.00–1.02), 0.099
Underlying C&R deaths	1.01 (0.95–1.08), 0.771	–	1.01 (0.99–1.02), 0.343	1.00 (0.99–1.01), 0.626
	–	1.05 (1.04–1.07), 0.000	1.01 (1.00–1.03), 0.037	1.00 (1.00–1.01), 0.166

*CI, confidence interval; RSV, respiratory syncytial virus; P&I, pneumonia and influenza; C&R, circulatory and respiratory. †Risk ratio estimates (95% confidence intervals) of each death category were adjusted for number of days in each month, linear and squared time trends, seasonal patterns, temperature, and relative humidity; –, risk factor was not included in the model.

Influenza B also had a significant effect on underlying C&R deaths (RR 1.01 for each 1% change in positive test results, 95% CI 1.00–1.03, p = 0.037) and all-cause deaths (1.01, 1.00–1.02, p = 0.008), but not on underlying P&I deaths (p = 0.878). RSV had no observable impact on all 3 death categories analyzed (RR range 1.00–1.01 for each 10% change in positive test results, p>0.099) (Table 4).

Next, we used the full model to quantify the excess deaths attributable to influenza throughout the year. For deaths from all causes, we estimated an annual mean of 588 influenza-associated deaths (Table 5), representing 3.8% of total deaths. The mean annual estimates of deaths from underlying P&I and C&R associated with influenza were 116 and 475, respectively, representing 6.5% and 5.8% of such deaths.

**Table 5:** Estimated influenza-associated excess deaths in Singapore, 1996–2003

Mortality outcome/age group (y)	Deaths (%) associated with influenza (95% CI)*	No. excess deaths per year (95% CI)	Excess mortality rate/100,000 person-years (95% CI)
All-cause deaths			
All ages	3.8 (2.5–5.0)	588 (396–782)	14.8 (9.8–19.8)
>65	4.2 (2.7–5.6)	421 (273–571)	167.8 (107.0–229.5)
20–64	2.3 (0.9–3.7)	114 (42–186)	4.2 (1.6–6.8)
Underlying pneumonia and influenza deaths			
All ages	6.5 (2.2–10.5)	116 (40–196)	2.9 (1.0–5.0)
>65	7.7 (3.5–11.7)	118 (50–189)	46.9 (20.3–74.6)
20–64	9.6 (3.0–15.7)	23 (7–39)	0.8 (0.2–1.4)
Underlying circulatory and respiratory deaths			
All ages	5.8 (4.0–7.5)	475 (324–629)	11.9 (8.3–15.7)
>65	6.2 (4.4–8.1)	390 (270–512)	155.4 (108.8–203.0)
20–64	4.6 (2.5–6.7)	88 (47–131)	3.2 (1.7–4.8)

*CI, confidence interval.

We observed that the proportion of influenza-associated deaths was higher among the elderly. The annual influenza-associated proportion of deaths from all causes was 11.3 times higher in persons age >65 years (167.8/100,000 person-years) than in the general population (14.8/100,000). For influenza-associated underlying P&I deaths, the annual death rate in those >65 years (46.9/100,000) was 16.2 times higher than those in the general population (2.9/100,000) (Table 5).

Table 6 compares the excess deaths observed in our study with that derived from studies in a subtropical and temperate country (*6**,**7*). While we observed a smaller overall impact for all ages than that reported in Hong Kong (*6*) and the United States (*7*), we noted a higher proportion of influenza-associated deaths among the elderly in Singapore.

**Table 6:** Annual influenza-associated deaths in Singapore, Hong Kong, and United States

Author	Country	Statistical method	Influenza-associated mortality rate/100,000 person-years		
			All-cause	Underlying pneumonia and influenza deaths	Underlying circulatory and respiratory deaths
Chow et al.	Singapore	Negative binomial regression model was used to estimate mortality outcomes. The model was developed by using monthly number of deaths and monthly proportion of positive influenza test results. Linear and nonlinear time trends, 3–4 pairs of seasonality variables, monthly mean temperature and relative humidity, and monthly proportion of positive respiratory syncytial virus (RSV) test results were included as covariates in the model.	All ages: 14.8	All ages: 2.9	All ages: 11.9
			>65 y: 167.8	>65 y: 46.9	>65 y: 155.4
Wong et al. (*6*)	Hong Kong	Poisson regression model was used to estimate mortality outcomes. The model was developed by using weekly number of deaths and weekly proportion of positive influenza test results. Dummy variables for each year, 2 pairs of seasonality variables, weekly mean temperature and relative humidity, and weekly proportion of positive RSV test results were included as covariates in the model.	All ages: 16.4	All ages: 4.1	All ages: 12.4
			>65 y: 136.1	>65 y: 39.3	>65 y: 102.0
Thompson et al. (*7*)	United States	Age-specific Poisson regression models were used to estimate mortality outcomes. Each model was developed by using weekly number of deaths for the specific age group and weekly proportion of positive influenza test results. Age-specific population size, linear and nonlinear time trends, 1 pair of seasonality variables, and weekly proportion of positive RSV test results were included as covariates in each model.	All ages: 19.6	All ages: 3.1	All ages: 13.8
			>65 y: 132.5	>65 y: 22.1	>65 y: 98.3

## Discussion

To our knowledge, our findings are the first to demonstrate that influenza activity is associated with excess deaths in a tropical country. Our estimates of annual influenza-associated all-cause deaths, underlying P&I deaths, and underlying C&R deaths in Singapore were 14.8, 2.9, and 11.9 per 100,000 person-years, respectively. This finding would translate to an estimated 588 deaths (3.8% of total deaths) due to influenza annually, which is comparable to the proportion of deaths observed in subtropical Hong Kong (*6*) and in the United States (*7*), a temperate country.

Our estimate of 46.9 underlying P&I deaths per 100,000 persons age >65 years each year is lower than the estimate of a local study (*21*). However, that study acknowledged a possible overestimation of the incidence of influenza in the elderly. Moreover, their estimates were based on the assumption that 40% of P&I deaths were associated with influenza, which was a figure derived from external data from temperate countries. This figure far exceeds our estimate of 6.5% of underlying P&I deaths attributable to influenza. In Hong Kong (*6*) and the United States (*7*), influenza-associated deaths represented 7.4% and 9.8% of underlying P&I deaths, respectively.

In Singapore, we observed that the influenza-associated proportion of deaths was highest in persons >65 years. Again, this finding is consistent with those in the United States where 90% of influenza-associated deaths occurred among the elderly (*7*). In this population, we estimated an annual number of excess deaths per 100,000 population of 167.8 of all-cause deaths, 46.9 deaths from underlying P&I, and 155.4 deaths from underlying C&R attributable to influenza.

In fact, our estimates for influenza-associated deaths in persons age >65 years were consistently higher than those in Hong Kong and United States, for all 3 mortality outcomes. A possible reason could be the use of influenza vaccines among vulnerable elderly is higher in the United States and Hong Kong than in Singapore. Influenza vaccination for all persons age >65 years is a well-established recommendation of the Advisory Committee on Immunization Practice (ACIP) in the United States (*22*). Vaccine coverage among elderly persons (>65 years) in the United States increased from 15% to 20% before 1980 to 65% in 2001 (*23*), and the national target of 60% coverage in this population has been achieved since 1997 (*24*). In Hong Kong, the use of vaccine has been limited (*25*). However, the vaccine has been recommended for institutionalized elderly since 1997, and the Department of Health has had a program to vaccinate this population since 1998 (*26*). In Singapore, influenza vaccine use has been low, and the mean annual quantity used in 2001–2002 was only ≈20,000 doses (Ministry of Health, Singapore, unpub. data). The number of persons age >65 averaged ≈250,000 during that period (*27*). Even if all 20,000 vaccine doses had been given to this group of persons, vaccination coverage in the elderly would not have exceeded 8% per year.

Annual influenza vaccination for persons age >65 years has been recommended since September 2003 in Singapore by the National Expert Committee on Immunization. Influenza vaccine efficacy for preventing death among people >65 years was estimated to be 68% (*28*). In a recent study, vaccine effectiveness in those >75 years of age was found to be even greater (*29*). However, such studies have yet to be conducted in the tropics. We recommend a follow-up study to estimate the impact of vaccination on influenza-associated deaths in this age group in Singapore.

With regard to influenza subtypes, we note that most seasons in the United States were dominated by influenza A (H3N2) virus (*30*); the greatest number of influenza-associated deaths were associated with influenza A (H3N2), followed by RSV, influenza B, and influenza A (H1N1) virus (*31*). Influenza A (H3N2) virus accounted for 60% and 77% of positive influenza isolates in the United States (*7*) and Hong Kong (*6*), respectively. Our findings were similar. Influenza A (H3N2) was the predominant virus subtype during our study period and had a consistently significant impact on all 3 categories of deaths. Although influenza B was noted to have significant effects on all-cause deaths and underlying C&R deaths, the magnitudes of RRs were relatively small (RR 1.00–1.01, for each 1% change in positive test results). In addition, influenza B virus did not have any significant and observable impact on underlying P&I deaths. We did not observe any significant impact from influenza A (H1N1) virus and RSV on all 3 outcomes.

One limitation of our study may have been that the effect of RSV could have been obscured when we analyzed data on all ages. This virus is known to predominately affect children <2 years of age (*8**–**10*), <5% of the population. However, this factor does not negate the main finding that influenza infections are associated with substantial disease in Singapore.

Studies suggest that global interhemispheric circulation of epidemics follows an irregular pathway with recurrent changes in the leading hemisphere (*32*). As a major travel hub with a high volume of travelers from both hemispheres, Singapore could be a sentinel for detecting changes in the circulating virus strain and contribute toward an understanding of influenza virus circulation pathways. The prevalence of influenza in Singapore illustrates the importance of improving worldwide coverage and quality of virologic and epidemiologic surveillance for influenza, as described in WHO's Global Agenda for Influenza Surveillance and Control (*33*).

Our findings have a few policy implications. First, they support the recent recommendation by the National Expert Committee on Immunization on annual influenza vaccination for elderly Singaporeans and for persons at high risk of having complications from influenza. Second, the finding that influenza infections account for substantial disease supports our continued investment in strengthening influenza surveillance in our country. Finally, the study provides justification for stockpiling antiviral drugs in our national influenza pandemic preparedness plan. An influenza pandemic can be expected to result in far higher attack and death rates (*34**,**35*) than currently observed. The extent of disease and economic impact caused by an influenza pandemic could be greatly reduced by the appropriate use of vaccines and antiviral drugs (*36*).

## Conclusion

In 2003, a new infectious disease, severe acute respiratory syndrome (SARS), emerged, which caused 238 cases and 33 deaths in Singapore (*37*). The SARS outbreak galvanized public health actions in surveillance and control. Surveillance and plans for containing a resurgence of SARS remain in place, in spite of the low risk of a recurrence. Influenza, in contrast, has caused an average of 588 excess deaths in Singapore annually. Influenza continues to cause an increasing amount of disease in Singapore, particularly in our rapidly aging population. However, available strategies in influenza prevention and control have yet to be optimized, largely because the true impact of influenza has been masked by the lack of a clear seasonal pattern in the tropics. The extent of the infection has remained largely unseen.

Our study is the first to show unequivocally that influenza has a significant impact on proportion of deaths in a tropical country like Singapore. The estimated excess deaths, while less than that observed in subtropical and temperate countries, still constitutes a substantial problem. As influenza-associated deaths are largely preventable through vaccination and the judicious use of antiviral drugs, our findings can influence the public health management of this disease.

## Appendix

We developed 6 negative binomial regression models to examine the relationships between proportion of deaths and the respiratory viruses, namely, influenza A virus, influenza B virus, and respiratory syncytial virus (RSV) (Table 3). The models were written as follows:

### Model 1

Monthly number of deaths = monthly proportion of influenza A + number of days in each month + linear time trend + squared time trend + 3–4 pairs of seasonality variables + monthly mean temperature + monthly mean relative humidity

### Model 2

Monthly number of deaths = monthly proportion of influenza B + number of days in each month + linear time trend + squared time trend + 3–4 pairs of seasonality variables + monthly mean temperature + monthly mean relative humidity

### Model 3

Monthly number of deaths = monthly proportion of RSV + number of days in each month + linear time trend + squared time trend + 3–4 pairs of seasonality variables + monthly mean temperature + monthly mean relative humidity

### Model 4

Monthly number of deaths = monthly proportion of influenza A + monthly proportion of influenza B + number of days in each month + linear time trend + squared time trend + 3–4 pairs of seasonality variables + monthly mean temperature + monthly mean relative humidity

### Model 5

Monthly number of deaths = monthly proportion of Influenza A + monthly proportion of RSV + number of days in each month + linear time trend + squared time trend + 3–4 pairs of seasonality variables + monthly mean temperature + monthly mean relative humidity

### Model 6

Monthly number of deaths = monthly proportion of influenza A + monthly proportion of influenza B + monthly proportion of RSV + number of days in each month + linear time trend + squared time trend + 3–4 pairs of seasonality variables + monthly mean temperature + monthly mean relative humidity
